# Prioritization of Quality Principles for Health Apps Using the Kano Model: Survey Study

**DOI:** 10.2196/26563

**Published:** 2022-01-11

**Authors:** Christin Malinka, Ute von Jan, Urs-Vito Albrecht

**Affiliations:** 1 Peter L Reichertz Institute for Medical Informatics TU Braunschweig and Hannover Medical School Hannover Germany; 2 Department of Digital Medicine Medical Faculty OWL Bielefeld University Bielefeld Germany

**Keywords:** Kano, quality principles, mobile apps, physicians, surveys and questionnaires, evaluation studies, mHealth, health apps

## Abstract

**Background:**

Health apps are often used without adequately taking aspects related to their quality under consideration. This may partially be due to inadequate awareness about necessary criteria and how to prioritize them when evaluating an app.

**Objective:**

The aim of this study was to introduce a method for prioritizing quality attributes in the mobile health context. To this end, physicians were asked about their assessment of nine app quality principles relevant in health contexts and their responses were used as a basis for designing a method for app prioritization. Ultimately, the goal was to aid in making better use of limited resources (eg, time) by assisting with the decision as to the specific quality principles that deserve priority in everyday medical practice and those that can be given lower priority, even in cases where the overall principles are rated similarly.

**Methods:**

A total of 9503 members of two German professional societies in the field of orthopedics were invited by email to participate in an anonymous online survey over a 1-month period. Participants were asked to rate a set of nine app quality principles using a Kano survey with functional and dysfunctional (ie, positively and negatively worded) questions. The evaluation was based on the work of Kano (baseline), supplemented by a self-designed approach.

**Results:**

Among the 9503 invited members, 382 completed relevant parts of the survey (return rate of 4.02%). These participants were equally and randomly assigned to two groups (test group and validation group, n=191 each). Demographic characteristics did not significantly differ between groups (all *P*>.05). Participants were predominantly male (328/382, 85.9%) and older than 40 years (290/382, 75.9%). Given similar ratings, common evaluation strategies for Kano surveys did not allow for conclusive prioritization of the principles, and the same was true when using the more elaborate approach of satisfaction and dissatisfaction indices following the work of Timko. Therefore, an extended, so-called “in-line-of-sight” method was developed and applied for this evaluation. Modified from the Timko method, this approach is based on a “point of view” (POV) metric, which generates a ranking coefficient. Although the principles were previously almost exclusively rated as *must-be* (with the exception of resource efficiency), which was not conducive to their prioritization, the new method applied from the *must-be* POV resulted in identical rankings for the test and validation groups: (1) legal conformity, (2) content validity, (3) risk adequacy, (4) practicality, (5) ethical soundness, (6) usability, (7) transparency, (8) technical adequacy, and (9) resource efficiency.

**Conclusions:**

Established survey methodologies based on the work of Kano predominantly seek to categorize the attributes to be evaluated. The methodology presented here is an interesting option for prioritization, and enables focusing on the most important criteria, thus saving valuable time when reviewing apps for use in the medical field, even with otherwise largely similar categorization results. The extent to which this approach is applicable beyond the scenario presented herein requires further investigation.

## Introduction

### Background

Independent of their proficiency with apps and the respective usage contexts, users are often unfamiliar with the intricacies of the specific aspects that are essential for recognizing an app’s quality. Even apps covering health contexts are often marketed without having been evaluated by experts, and with only minimally relevant and reliant information being provided (eg, regarding scientific studies [1,2]). Thus, for end users, making an informed decision about whether or not to use an app is not an easy task, independent of whether they are health care professionals, patients with chronic conditions, or even laypeople with a more generic interest in health apps.

There are numerous, more or less elaborate, tools, norms, and lists of quality criteria that either target developers or aim at aiding those interested in an app in their decision process (eg, [3-8]), and many of the aspects they cover overlap. However, even if interested parties are aware of these approaches, if a quick assessment is desired, these approaches may sometimes be seen as going too far or being too complex. Both the paucity of readily available information or expert assessments [1,9] in identifying apps that can be recognized as trustworthy, as well as the difficulty in identifying suitable criteria for an initial and independent assessment, can mean that apps often fail to realize the potential attributed to them for medical care and prevention [10-13]. Checklists that interested users may apply to apps (eg, [4,14,15]) often target careful curation of a list of apps for later use, but may be too extensive for practical application and quick assessments in everyday medical practice. It may therefore be helpful to develop and apply a process for identifying a subset of criteria or quality principles listed in such tools considered to be particularly relevant for a specific target group, which may be achieved by means of prioritization.

As a foundation for this study, we used nine basic quality principles for health apps that were previously compiled [16,17] and evaluated [18,19] in a multistep process: (1) practicality, (2) risk adequacy, (3) ethical soundness, (4) legal conformity, (5) content validity, (6) technical adequacy, (7) usability, (8) resource efficiency, and (9) transparency. Participants in both of the aforementioned evaluation studies were first requested to provide initial assessments regarding the perceived relevance of these principles. They were then provided with applied app store descriptions and asked to determine whether they deemed the textual information sufficient to satisfy the above principles. Subsequently, they were asked to apply 25 questions operationalizing the nine principles to the same store descriptions. Between each of the steps, they were asked whether or not they would consider using the respective app based on the available information. During the course of these studies [18,19], as participants familiarized themselves with the quality criteria, they were able to make a more confident, but increasingly critical, assessments of the apps based on the available information.

These previous studies with medical students [18] and members of the German Society for Internal Medicine [19] showed that the participants predominantly perceived all nine of the above quality principles as important. For both studies, the data were evaluated using two (randomly assigned and equally sized) test and validation groups [18,19]. Although there were no significant differences in the answers obtained for the nine principles between the two groups, solely based on assigned relevance, rankings (and thus any prioritizations based on them) would have differed [18] between the groups as well as between the two studies. Apart from slightly lower relevance ratings for resource efficiency, all other quality principles were seen as either “important” or “very important”; however, owing to their closeness with respect to the ratings, any order of the principles based on these ratings seemed to have been influenced by statistical noise rather than sound calculations. Nevertheless, in both of the aforementioned studies [18,19], some participants expressed fear that the application of even these few principles would be too time-consuming for use in an everyday care context. As even the relevance-related questions for the criteria that were asked in these studies did not allow for their ranking, we therefore aimed to establish a method that would meet the demand for a better focus on quality aspects for mobile health (mHealth) apps that would be perceived as particularly relevant in the community.

### Hypotheses

We hypothesized that methods established to assess product attributes in marketing-related research might also be suitable for categorizing quality attributes for mHealth apps. We tested this hypothesis based on an exemplary Kano survey related to the nine aforementioned quality principles. In this type of survey, questions are implemented based on a model developed by Noriaki Kano in the 1970s and 1980s. The “Kano model” is often used in the context of marketing or for refining products, specifically with regard to customer satisfaction with a product’s features in mind [20]. As Kano noted, there need not be a linear relationship between satisfaction or dissatisfaction and the fulfillment of a need [21]; thus, to be able to nevertheless assess a product, he proposed using so-called “functional” and “dysfunctional” questions that not only assess a participant’s opinion about a feature being available but also about it not being provided.

On its own, if successful at all, such a Kano survey–based categorization can only provide a rough prioritization at best, based on ranking the categories according to their fitness for the question at hand. As this approach may fail in cases where the attributes under consideration are rated similarly, we established our second hypothesis that it should be possible to nevertheless prioritize the product attributes studied (in our case, the nine quality principles) by developing and applying an extended method on the basis of the data collected.

### Objectives

This study builds upon the foundation laid by previous studies in the health app quality context. This work was motivated by interest to find and apply a method that helps to more finely differentiate between a chosen set of quality attributes to be used in such a setting. As indicated above, although there are a variety of tools for this task or lists of quality principles for different app types in the mHealth domain, there are voices lamenting that despite these tools being academically sound, applying them in a real-world setting or for a large number of apps may be too tedious [22].

In our evaluation, the proposed method was applied to the nine predefined health app quality principles to determine whether it is feasible to determine an adequate and stable ranking of such criteria to be used for prioritization in facilitating app assessments should the need arise.

### Basic Design of the Study

Our approach is based on a group of popular techniques for classifying quality attributes that are often used in decision-making processes in the areas of marketing, management, or even a product’s design phase [23] if a decision is to be made about which (planned or existing) attributes of a product elicit customer satisfaction (and should thus be used or further investigated for a product) or dissatisfaction (making them superfluous or even counterproductive for the product’s success). Following this line of thought, we used a survey design based on Kano’s model of attractive quality for classifying quality attributes (originally published in Japanese [20] and subsequently in English [24]), and applied various more elaborate evaluation techniques as specified in the literature (eg, those proposed by Timko as cited in Berger et al [25]) to the acquired data.

Using the Kano survey data and available evaluation methods, it may be conceivable to find sufficiently differing categorizations of the quality principles that allow for selecting a particularly relevant subset of principles based on their assigned (Kano or derived) category, whereas principles in lower-ranking or less-desirable categories are treated as deferred or are even removed from further consideration. As applied to the nine quality principles, we suspected that even if the principles are largely seen as similarly important, some might be viewed as more attractive, essential, or indifferent than others. Based on a per-category ranking (depending on the perceived relevance of the categories for the use case), we deemed it possible to determine at least a partial prioritization.

As the first idea was unfortunately quickly disproved due to the largely similar categorizations of the nine principles based on the acquired survey data, as a second approach, we tried to better take into account to what degree a product’s attributes, or in our case the app quality principles, contribute to (customer) satisfaction or dissatisfaction, specifically based on the work proposed by Timko in Berger et al [25]. Our assumption was that by appropriately taking both the numeric values for satisfaction as well as dissatisfaction into account, it should be possible to determine a numeric representation in the form of a ranking coefficient (eg, using a ratio of the two values or similar approaches) that could lay the foundation for finding a relatively stable means for prioritization of app quality principles based on this value.

## Methods

### Data Acquisition

#### Implementation

Data collection for the study took place in the form of an anonymous and data protection–compliant online survey, implemented using the SoSci Survey [26] installation provided at Hannover Medical School. The survey was open for 1 month (between December 2, 2019, and January 2, 2020), and using the mailing lists of both the German Society for Orthopedics and Trauma Surgery (DGOU) and the Orthopedics and Trauma Surgery Professional Association (BVOU); a total of 9503 members of these societies were invited to participate.

Prior to sending the survey invitation, the study was reviewed by the Ethics Committee of Hannover Medical School (application number 8746_BO_K_2019). In the vote dated November 4, 2019, no ethical or legal objections were raised.

#### Structure of the Survey

The actual survey itself was conducted in two parts. The first part contained questions about the German Digital Healthcare Act (DVG [27]) that, at the time of the survey, had recently been ratified. Participants were presented with questions about their familiarity with this act, their opinions about its coverage, and whether they were at all considering making use of the possibility to prescribe health apps based on the processes specified in the DVG. The data corresponding to this part of the survey were previously evaluated and published [28].

To acquire demographic data, those responding to the survey were asked questions related to age and gender, as well as about their work history and environment (how long they had been working; their current function; and whether they were working in private practice, at a clinic, or another institution). To allow a basic assessment about their familiarity with mHealth, they were also asked about their private and work-related usage of mHealth apps, and whether any patients asked them either about specific health apps or about a recommendation for a health app. However, the demographic data are only presented to describe the participating physicians. Apart from exemplary calculations given in the Discussion, these data were not part of the analyses presented in this paper.

The work presented herein specifically deals with the second part of the survey. As mentioned in the Introduction, a predefined set of nine quality principles (practicality, risk adequacy, ethical soundness, legal conformity, content validity, technical adequacy, usability, resource efficiency, and transparency) was employed as a basis for the evaluation. The set of quality principles has previously been published [16, 17] along with their evaluations [18,19].

In the context of the work presented here, following Kano’s method, for each of the nine quality principles, the participants were presented with a set of so-called functional and dysfunctional questions (see Table 1). Answer options for both types of questions were “I would be very pleased,” “I’d expect this,” “I don’t care,” “I could accept that,” and “That would really bother me.”

**Table 1:** Quality principles with the corresponding questions (translated from the original German-language version) for functional and dysfunctional aspects, as required by the Kano model.

Principle	Functional question	Dysfunctional question
Practicality	What would you say if apps could be used for the intended purpose?	What would you say if apps could not be used for the intended purpose?
Risk adequacy	What would you say if apps did not pose a disproportionate health, social, or economic risk to users?	What would you say if apps posed disproportionate health, social, or economic risks to users?
Ethical soundness	What would you say if discrimination and stigmatization were avoided when developing, offering, and using apps?	What would you say if discrimination or stigmatization were not avoided when developing, offering, operating, and using apps?
Legal conformity	What would you say if apps were compliant with data protection regulations as well as professional and health regulations?	What would you say if apps failed to comply with data protection, professional, or health regulations?
Content validity	What would you say if the content used in apps was valid and trustworthy?	What would you say if the content used in apps was not valid or not trustworthy?
Technical adequacy	What would you say if apps were easy to maintain and could be used independent of a specific platform?	What would you say if apps were hard to maintain or could not be used independent of a specific platform?
Usability	What would you say if apps were designed and implemented according to the requirements of the target group(s)?	What would you say if apps were not designed and implemented to meet the needs of the target group(s)?
Resource efficiency	What would you say if apps were to use resources such as battery and computing power efficiently?	What would you say if apps made only inefficient use of resources such as battery or computing power?
Transparency	What would you say if apps provided transparent information about inherent quality features?	What would you say if apps did not provide transparent information about inherent quality characteristics?

In addition to the functional and dysfunctional questions, the participants were also asked to rate the perceived relevance for each of the nine principles (Table 2). In this case, answers could be given using a 5-point scale: “very important,” “important,” “neutral,” “less important,” and “unimportant.”

For each quality principle, the “functional” question was always presented first, followed by the “dysfunctional” question, and that for relevance. However, for each participant, the order in which the questions were shown was randomly assigned to alleviate bias based on an attribute’s position in the list.

**Table 2:** Questions regarding the relevance for each of the nine quality principles (translated from the original German version).

Principle	Perceived relevance
Practicality	How important is it to you that apps can be used for the intended purpose?
Risk adequacy	How important is it to you that apps are low risk in terms of health, social, or economic risks?
Ethical soundness	How important is it to you to avoid discrimination and stigmatization when developing, offering, operating, and using apps?
Legal conformity	How important is it to you that data protection, professional, and health regulations are respected in apps?
Content validity	How important is the validity and trustworthiness of the health-related content presented and used in an app to you?
Technical adequacy	How important are easy maintainability and platform-independent or cross-platform usability of apps to you?
Usability	How important is the target group–oriented design and operation of apps to you?
Resource efficiency	How important to you is the efficient use of resources through apps, for example in terms of battery and computing power?
Transparency	How important is it to you that apps provide transparent information about inherent quality features?

#### Categorization of Answers According to Kano

Using the Kano model, based on the answers given for both functional and dysfunctional questions (see Table 3), a product’s features can be categorized as *attractive (A)*, if its presence leads to satisfaction but there is no (additional) dissatisfaction if it is missing [25]; *must-be (M)*, if the respective feature is deemed essential (ie, if it does not improve satisfaction if available, but leads to extreme dissatisfaction if missing) [29]; *one-dimensional (O)*, also referred to as the *performance (P)* category in the literature, if both availability and lack of the feature cause satisfaction and dissatisfaction, respectively [25], thus representing a feature that customers explicitly demand; *indifferent (I)*, if the feature (or the lack thereof) influences neither satisfaction nor dissatisfaction, thus being ideal for elimination if a reduction in overhead is desired [30]; *reverse (R)*, if dissatisfaction is caused if the feature is available and satisfaction if it is missing; and *questionable (Q)* if the answers given to the functional and dysfunctional questions are in contradiction [25] (eg, if both answers are specified as “I would be very pleased”).

**Table 3:** Assignment of answers to various categories to both functional and dysfunctional questions (based on [25]) and representation of answer pairs where one or both answers are missing.

Answers to functional questions	Answers to dysfunctional questions					
	I would be very pleased	I’d expect this	I don’t care	I could accept that	That would really bother me	No answer given
I would be very pleased	Q^a^	A^b^	A	A	P^c^	—^d^
I’d expect this	R^e^	Q	I^f^	I	M^g^	—
I don’t care	R	I	I	I	M	—
I could accept that	R	I	I	Q	M	—
That would really bother me	R	R	R	R	Q	—
No answer given	—	—	—	—	—	—

^a^Q: questionable.

^b^A: attractive.

^c^P: performance (one-dimensional).

^d^Not applicable.

^e^R: reverse.

^f^I: indifferent.

^g^M: must-be.

Both *the reverse and questionable* categories may, for example, be due to inadequate wording of the questions employed in the survey or side effects from other (not necessarily easily explainable) factors that impact the answers. Especially for the questionable category, the answers given may also indicate that a participant was (for whatever reason) unwilling to answer in a sensible manner.

### Evaluation Strategies

For each of the nine quality principles, the answers provided by the participants for the functional and dysfunctional question pairs were then categorized based on Table 3, and the frequency that each category was assigned to each attribute was calculated. These counts were then used for further evaluation. As described previously [25,31], there are several strategies that can be applied for this task.

One approach is to determine the category for a feature based on its greatest frequency. Alternatively, an if-then–based approach can be adopted: if (*P*+*A*+*M*)>(*I*+*R*+*Q*), the category that corresponds to the maximum count for *performance*, *attractive*, or *must-be* is used; however, if (*P*+*A*+*M*)<(*I*+*R*+*Q*), the category corresponding to the maximum of *indifferent*, *reverse*, or *questionable* as the category assigned to the feature under consideration is used.

Both of these approaches work best if those surveyed are somewhat consistent in their answers for a specific feature, or at least show a clear tendency toward a specific category for that feature. However, these approaches do not work quite as well if the responses are distributed more evenly across several categories such as *attractive*, *performance*, *must-be*, and *indifferent*. Moreover, if different features elicit similar responses, it may be difficult to discriminate between them. This may hamper the usefulness of the approach in the context of categorization.

Timko (cited in [25]) proposed an additional method, as he noted that based on the aforementioned mode statistic, the results may seem somewhat skewed. For example, for two features with only *attractive* and *indifferent* ratings, albeit one with a 90-to-10 *attractive-*to-*indifferen*t ratio and the other with only a 60-to-40 *attractive-*to-*indifferent* ratio, the assigned category will be *attractive* for both. Thus, a third method tries to alleviate these disadvantages.

This method uses the previously obtained counts to calculate two distinct values: one representing the relative value of meeting a customer requirement (namely, “what if we’re better” in contrast to a competitor) and the other representing the relative cost of not meeting the customer requirement (ie, worse than the competition). The two values, as defined in Berger et al [25], are calculated as follows:


Better = (*A*+*P)*/(*A*+*O*+*M*+*I)*, with 0 ≤ Better ≤ 1

Worse = –(*O*+*M)*/(*A*+*O*+*M*+*I)*, with –1 ≤ Worse ≤ 0

On average, satisfaction will increase for *attractive* and *one-dimensional* (*performance*) attributes, which is why, in the literature, “Better” is also often denoted as the satisfaction index [32,33], and satisfaction decreases if *one-dimensional* and *must-be* elements are not adequately represented. For this reason, *“Worse”* is often called the dissatisfaction index [32,33]. Both *questionable* as well as *reverse* answers are ignored in Timko’s approach, but nevertheless, the calculations do respect a possible spread of the attributes under consideration over the different categories.

**Figure 1:**
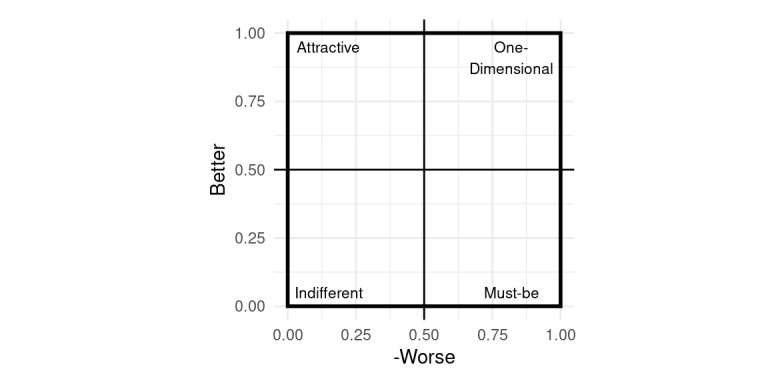
Two-dimensional representation of Worse-Better pairings for the Kano quality categories [25]. For easier interpretation, Worse is shown with its absolute value.

The Worse-Better pairing for calculated attributes can be plotted on a two-dimensional and easy-to-interpret graph. Commonly, the values for each attribute are additionally multiplied by the average relevance the participants assign to each attribute to improve discrimination between value pairs for features located in direct vicinity to each other. According to Timko, when deciding which attributes to keep or to omit, one should choose those for which satisfaction (ie, the Better score) is higher, since they add more to customer satisfaction, whereas on the Worse axis, one should aim for more negative values, as they prevent dissatisfaction [25] (Figure 1).

### Designing an Improved Methodology for Prioritization

Discussions among the authors led to the conclusion that established methods such as those described above were suffering from only being able to assign broadly defined categories to the attributes under consideration, without allowing for a more granular consideration that actually respects the relative location of the attributes under consideration. This is particularly relevant when the attributes to be compared (represented by their Worse and Better coordinates) are (predominantly) located in one of the four quadrants and are therefore assigned to the same category (ie, *indifferent*, *must-be*, *attractive*, or *one-dimensional*). With this in mind, we designed an “in-line-of-sight” method that allows for rankings depending on different points of view on the coordinate system.

This new approach makes it possible to establish a reference to the proximity of an attribute’s (or quality principle’s) coordinate points to the respective outermost corner (corresponding to the point most clearly representing the quadrant), and further respects their relative positions for obtaining the ranking.

This approach will now be explained in more detail by way of an example, using the *must-be* quadrant as a point of reference. Starting from the outermost point of this category, denoted by the coordinates (*Worse_I_*=–1, *Better_I_*=0), for each attribute (or quality principle), the Euclidean distance between this point and the respective coordinate is first calculated. An increasing distance to the *must-be* corner represents a greater proximity to one of the three other categories (and is, as such, less desirable).

**Figure 2:**
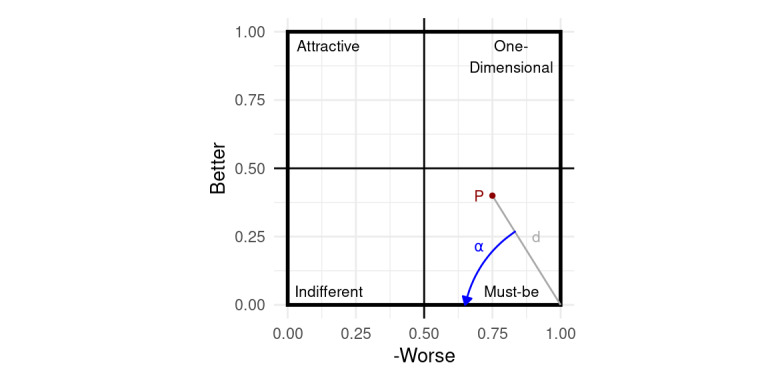
Angle (α) and distance (d) for a point (P) located in the must-be corner, as employed in the in-line-of-sight method (seen from the must-be corner).

For further improved differentiation between quality principles, even in the case of (almost) identical Euclidean distances, an angle is then determined based on the chosen secondary ranking strategy. In our example (and all further calculations shown in this paper), we decided to prefer points with less pronounced Worse values (ie, those that have less potential for causing dissatisfaction according to Timko). For this purpose, we chose to calculate an offset based on the angle (denoted by *α*) between the x-axis of the coordinate system and the line defined by the corner point’s coordinate *p*=(–1,0) as well as the respective quality principle’s *q=(–Worse_I_, Better_I_*) coordinate (see Figure 2). As *α* is only supposed to aid with differentiation between points with similar distance values, it needs to be rescaled to an appropriate value range. First, *α* is divided by the maximum possible angle (ie, 90°) and then multiplied with 0.05×2≈0 (representing 5% of the maximum possible distance of the square root of 2 in the coordinate system). The distance and adapted angle value are then summarized (hereinafter referred to as the ranking coefficient *f*), and the resulting value for *f* is then used for ranking the quality principles according to ascending order as follows: 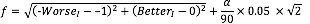



For simplification, as the plots use an inverted x-axis for representing the Worse value, all statements (as well as the angle calculations) concerning the left- or right-hand location of any point or axis mentioned in relation to the coordinate system refer to this inverted plot. For the other three quadrants, if necessary, rankings may be performed in a similar manner.

### Statistics Tools

The R language and environment for statistical computing, version 4.0, was used for all evaluations, along with accompanying packages such as dplyr, ggplot2, arsenal, and others [34-36].

## Results

### Data

Of those who answered our survey, only 382 actually completed all of its parts, and were thus included in the evaluation presented here. This corresponds to a return rate of 4.02% of the 9503 potential participants.

Using the sample_frac function provided by the dplyr package [34], the available participants were randomly assigned to the test (group A, n=191) and validation (group B, n=191) groups.

### Baseline Demographics of the Participants

To rule out differences between the two groups due to demographic factors, these were first compared. There were no statistically significant differences between the groups with respect to baseline demographics (*P*>.05 for all factors, see Table 4). Overall, the participants were predominantly male and older than 40 years (290/382, 75.9%). In line with the age structure, over three-quarters of the participants had a work experience of more than 10 years (288/328, 75.4%; excluding retirees, 19/328, 5.8%) and were working in higher-level functions (attendings, chiefs, or specialists in private practice; 284/382, 74.3%). The majority of participants worked in a hospital setting (acute care or university hospital; 232/382, 60.7%). As we had only surveyed members of two German orthopedic societies, the proportion of those who were not active in Germany was low, as expected (10/382, 2.6%).

Although the participants overwhelmingly stated that they were highly interested or interested in digital technology (316/382, 82.7%), this was not mirrored by the proportion of those admitting to app use in private or work settings. Only slightly over one-fifth of those participating had already been asked by patients about a specific app or about recommending an app (see Table 4 for full data).

**Table 4:** Base demographics for all participants and for those assigned to the test group (A) and validation group (B).

Characteristic			Group A (n=191), n (%)		Group B (n=191), n (%)		Total (N=382), n (%)		*P* value^a^	
**Age group (years)**										.87
	21-30	9 (4.7)		7 (3.7)		16 (4.2)				
	31-40	34 (17.8)		42 (22.0)		76 (19.9)				
	41-50	46 (24.1)		44 (23.0)		90 (23.6)				
	51-60	62 (32.5)		59 (30.9)		121 (31.7)				
	>60	40 (20.9)		39 (20.4)		79 (20.7)				
**Gender**										.38
	Female	24 (12.6)		30 (15.7)		54 (14.1)				
	Male	167 (87.4)		161 (84.3)		328 (85.9)				
**Work experience**										.93
	Not yet working	2 (1.0)		1 (0.5)		3 (0.8)				
	<1 year	2 (1.0)		2 (1.0)		4 (1.0)				
	1-5 years	10 (5.2)		14 (7.3)		24 (6.3)				
	6-10 years	19 (9.9)		25 (13.1)		44 (11.5)				
	11-20 years	50 (26.2)		44 (23.0)		94 (24.6)				
	21-30 years	54 (28.3)		50 (26.2)		104 (27.2)				
	>30 years	44 (23.0)		46 (24.1)		90 (23.6)				
	Retired	10 (5.2)		9 (4.7)		19 (5.0)				
**Professional level**										.75
	Student	1 (0.5)		0 (0.0)		1 (0.3)				
	In training/resident	23 (12.0)		25 (13.1)		48 (12.6)				
	Attending	60 (31.4)		52 (27.2)		112 (29.3)				
	Chief	38 (19.9)		39 (20.4)		77 (20.2)				
	Specialist (private practice)	47 (24.6)		48 (25.1)		95 (24.9)				
	Other	21 (11.0)		27 (14.1)		48 (12.6)				
	Not answered	1 (0.5)		0 (0.0)		1 (0.3)				
**Work setting**										.49
	Acute care: standard care level	63 (33.0)		50 (26.2)		113 (29.6)				
	Acute care: maximum care level	32 (16.8)		37 (19.4)		69 (18.1)				
	University hospital	21 (11.0)		29 (15.2)		50 (13.1)				
	Rehabilitation center	8 (4.2)		7 (3.7)		15 (3.9)				
	Medical care center	6 (3.1)		9 (4.7)		15 (3.9)				
	Private practice	40 (20.9)		44 (23.0)		84 (22.0)				
	Other	21 (11.0)		14 (7.3)		35 (9.2)				
	Not answered	0 (0.0)		1 (0.5)		1 (0.3)				
**Geographic location^b^**										.26
	Germany	187 (98.9)		183 (95.8)		370 (97.4)				
	Austria	0 (0.0)		2 (1.0)		2 (0.5)				
	Switzerland	2 (1.1)		3 (1.6)		5 (1.3)				
	Other: European Union	0 (0.0)		2 (1.0)		2 (0.5)				
	Other: not yet listed	0 (0.0)		1 (0.5)		1 (0.3)				
**Interest in digital technology**										.71
	Highly interested	76 (39.8)		81 (42.4)		157 (41.1)				
	Interested	84 (44.0)		75 (39.3)		159 (41.6)				
	Neutral	19 (9.9)		25 (13.1)		44 (11.5)				
	Less interested	8 (4.2)		8 (4.2)		16 (4.2)				
	Not interested	4 (2.1)		2 (1.0)		6 (1.6)				
**Uses apps in private settings**										.92
	Yes	69 (36.1)		70 (36.6)		139 (36.4)				
	No	122 (63.9)		121 (63.4)		243 (63.6)				
**Uses apps for work**										.29
	Yes	63 (33.0)		73 (38.2)		136 (35.6)				
	No	128 (67.0)		118 (61.8)		246 (64.4)				
**Been asked about an app/recommendation**										>.99
	Yes	43 (22.5)		43 (22.5)		86 (22.5)				
	No	148 (77.5)		148 (77.5)		296 (7.5)				

^a^Pearson *χ^2^* test.

^b^Not answered: group A, n=2.

### Data Evaluation

#### Descriptive Evaluation of the Survey Results

**Figure 3:**
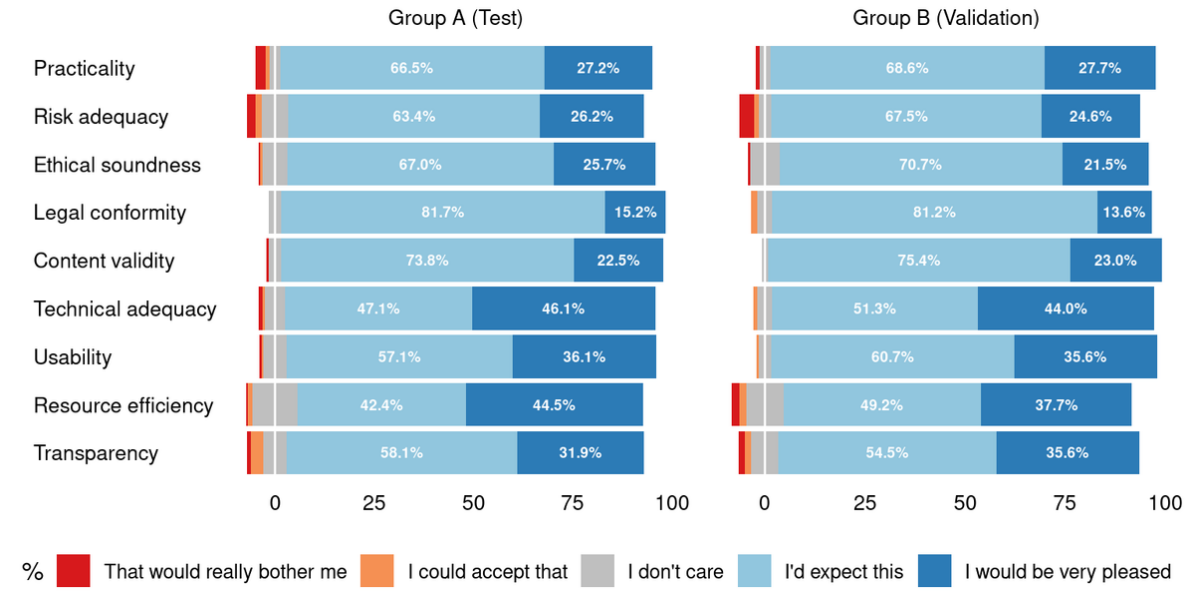
Distribution of answers for the functional questions. For legibility reasons, smaller values are not printed (see Multimedia Appendix 1 for the complete list of values).

**Figure 4:**
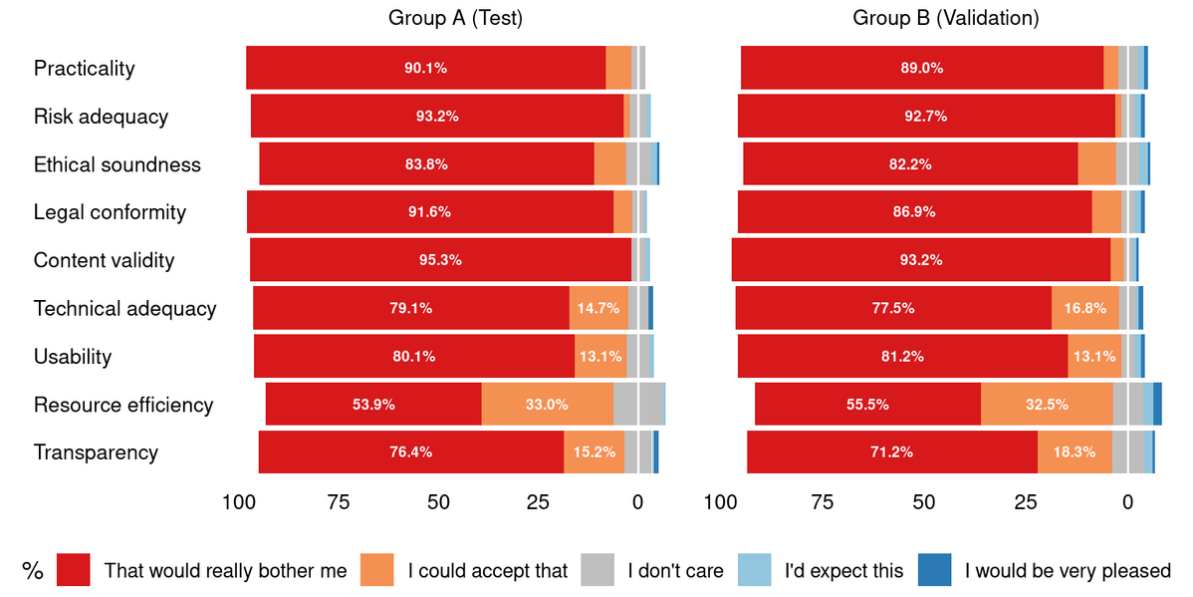
Distribution of answers for the dysfunctional questions. For legibility reasons, smaller values are not printed (see Multimedia Appendix 1 for the complete list of values).

**Figure 5:**
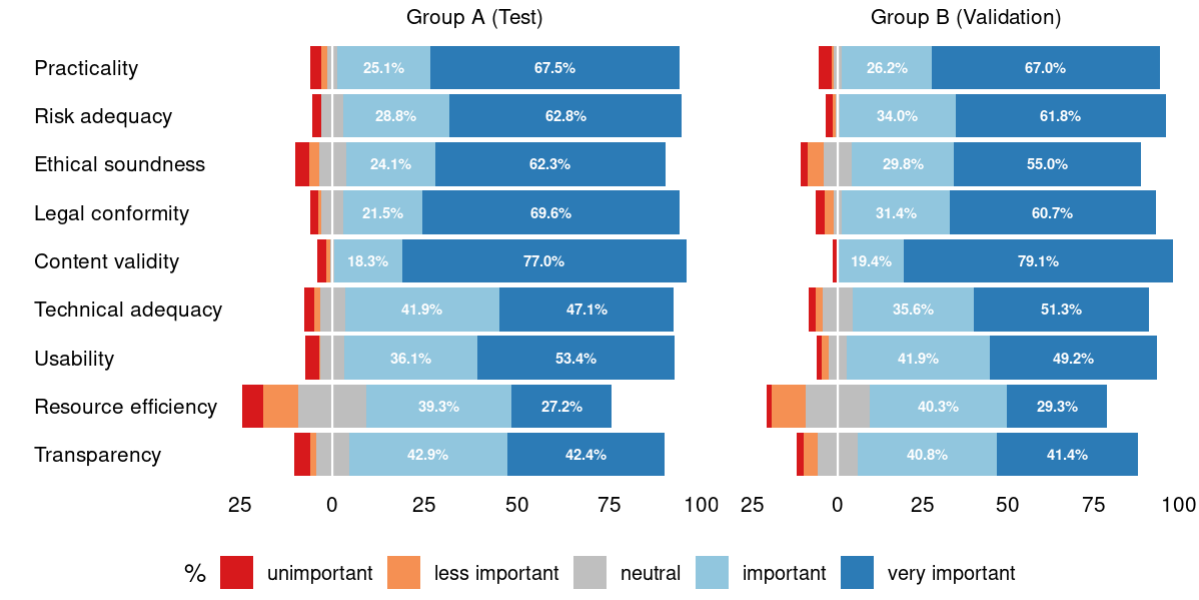
Ratings for relevance of the nine quality principles, as perceived by the participants. For legibility reasons, smaller values are not printed (see Multimedia Appendix 1 for the complete list of values).

Similar to the participants’ demographics, in the Kano-based questionnaire, there were no statistically significant differences between the training and validation groups with respect to answers given for the functional and dysfunctional questions, as well as the perceived relevance for the nine app quality criteria (see Figures 3, 4, and 5; for more detailed counts, proportions, and *P* values for the available answers, see Multimedia Appendix 1, Tables S1-S3).

#### Categorization According to Kano

Using Kano’s basic evaluation described in the “Evaluation Strategies Applied” subsection within the Methods, namely choosing the category with the largest number of counts as that to assign to each quality principle, the nine evaluated quality principles were exclusively categorized as *must-be* (see Table 5). This gives all attributes equal impact, which made it impossible to prioritize certain quality principles as desired, despite differences in ratings.

**Table 5:** Categorization of the answers for the functional and dysfunctional questions related to the nine quality principles, based on the category with the maximum count.

Quality principle	Test group, A (n=191)							Validation group, B (n=191)							
	M^a^	P^b^	A^c^	I^d^	R^e^	Q^f^	Category	M		P	A	I	R	Q	Category
Practicality	127	42	10	7	2	3	M	122		48	4	12	2	3	M
Risk adequacy	127	48	2	9	1	4	M	127		46	0	7	3	8	M
Ethical soundness	120	40	8	19	1	3	M	123		33	7	23	0	5	M
Legal conformity	148	27	2	13	0	1	M	146		20	5	15	1	4	M
Content validity	139	42	1	7	0	2	M	140		38	5	6	0	2	M
Technical adequacy	83	68	20	18	2	0	M	89		59	24	16	1	2	M
Usability	103	49	20	17	0	2	M	105		50	16	15	0	5	M
Resource efficiency	63	40	45	40	1	2	M	69		37	34	40	6	5	M
Transparency	103	43	18	23	3	1	M	89		45	22	27	1	7	M

^a^M: must-be.

^b^P: performance.

^c^A: attractive.

^d^I: indifferent.

^e^R: reverse.

^f^Q: questionable.

For example, for resource efficiency, less than half as many answer pairs were categorized under *must-be* compared with those for content validity (Group A: 63 vs 139 or 45.3%; Group B: 69 vs 140 or 49.3%); nevertheless, both principles were still equally categorized as *must-be*.

#### If-Then–Based Approach

The situation did not improve when employing the if-then approach; the results were equivalent to those shown in Table 5.

#### Timko Approach

**Figure 6:**
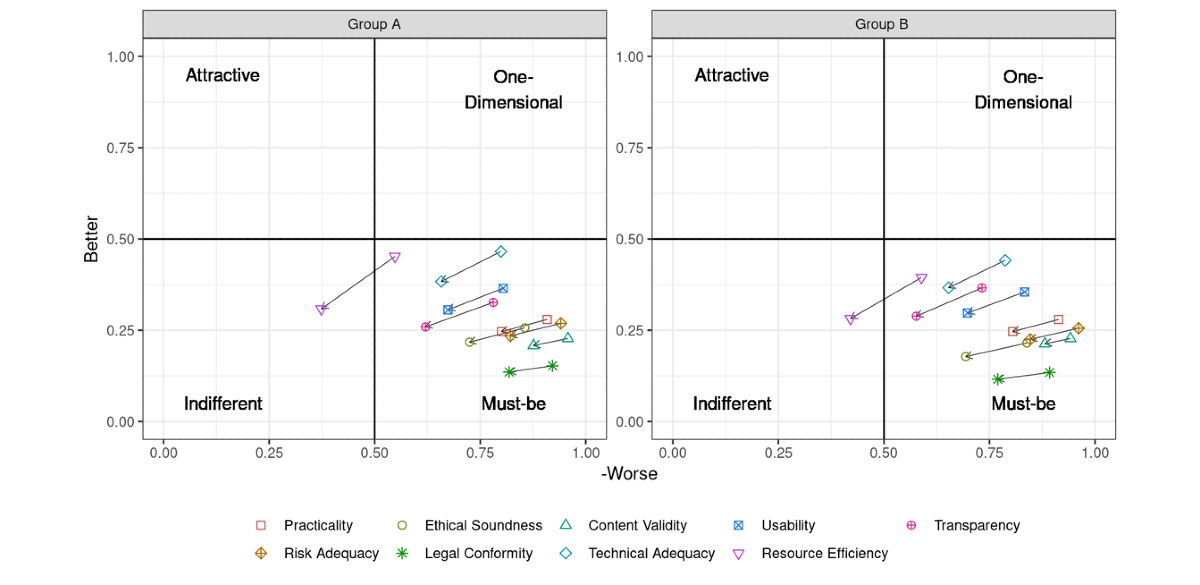
Better and Worse pairings for the training (Group A) and validation (Group B) groups, plotted with and without the average value for perceived importance. The arrows represent the corresponding coordinate shift from the original values to those factoring in the perceived importance for each quality principle.

Even using the method proposed by Timko [25], with or without using the average values for perceived importance, the situation only changed marginally, as shown in Table 6 and Figure 6. Visually, the value pairs were still in close vicinity to each other. Without factoring in perceived relevance, all values firmly remained categorized as *must-be*; only when accounting for relevance, one quality principle, specifically resource efficiency, showed a categorization change from *must-be* to *indifferent*. Apart from this principle (which, now being rated *indifferent* is deemed to be of less importance), prioritization of the remaining attributes was elusive, despite apparent (visual and numeric) differences.

**Table 6:** Better and Worse values without (denoted by a subscripted N) and with factoring in the average value of perceived relevance (or importance, denoted by a subscripted I) for each principle.

Quality principle	Group A						Group B				
	Better_N_	Worse_N_	Importance	Better_I_	Worse_I_	Better_N_		Worse_N_	Importance	Better_I_	Worse_I_
Practicality	0.28	–0.91	0.88	0.25	–0.80	0.28		–0.91	0.88	0.25	–0.81
Risk adequacy	0.27	–0.94	0.87	0.23	–0.82	0.26		–0.96	0.88	0.23	–0.85
Ethical soundness	0.26	–0.86	0.85	0.22	–0.72	0.22		–0.84	0.83	0.18	–0.69
Legal conformity	0.15	–0.92	0.89	0.14	–0.82	0.13		–0.89	0.86	0.12	–0.77
Content validity	0.23	–0.96	0.91	0.21	–0.88	0.23		–0.94	0.94	0.21	–0.88
Technical adequacy	0.47	–0.80	0.82	0.38	–0.66	0.44		–0.79	0.83	0.37	–0.65
Usability	0.37	–0.80	0.84	0.31	–0.67	0.35		–0.83	0.84	0.30	–0.70
Resource efficiency	0.45	–0.55	0.68	0.31	–0.37	0.39		–0.59	0.71	0.28	–0.42
Transparency	0.33	–0.78	0.79	0.26	–0.62	0.37		–0.73	0.79	0.29	–0.58

#### In-Line-of-Sight Method

Table 7 shows the rankings for both groups based on the *must-be* quadrant, as this is where the attributes predominantly clustered. Angles were calculated in the direction of the *one-dimensional* (*performance*) category.

The distances between Better-Worse pairings for both groups (ie, the distance between the two groups) only differed insignificantly: they always remained below 5% the maximum possible distance within the coordinate square (ie, 0.05×[(0,0),(–1,1)]=0.05×√2≈0.05×1.14142≈0.0707).

Based on the described method, the ranking for the quality principles was identical for both groups, with legal conformity ranked first, followed by content validity, risk adequacy, practicality, ethical soundness, usability, transparency, technical adequacy, and finally, resource efficiency.

**Table 7:** Ranking the quality principles based on distance to the must-be corner and angle toward the right-most boundary.

Quality principle	Coordinate distance between groups	Group A (test group)					Group B (validation group)			
		Distance, *d*	Angle, *α*	Ranking coefficient, *f*	Rank	Distance, *d*		Angle, *α*	Ranking coefficient, *f*	Rank
Practicality	0.00	0.32	51	0.36	4	0.31		52	0.35	4
Risk adequacy	0.03	0.29	53	0.34	3	0.27		56	0.32	3
Ethical soundness	0.05	0.35	38	0.38	5	0.35		30	0.38	5
Legal conformity	0.05	0.23	37	0.26	1	0.26		27	0.28	1
Content validity	0.01	0.24	59	0.29	2	0.24		61	0.29	2
Technical adequacy	0.02	0.51	48	0.55	8	0.50		47	0.54	8
Usability	0.03	0.45	43	0.48	6	0.42		45	0.46	6
Resource efficiency	0.05	0.70	26	0.72	9	0.64		26	0.66	9
Transparency	0.05	0.46	34	0.49	7	0.51		34	0.54	7

#### Gender Influence

**Figure 7:**
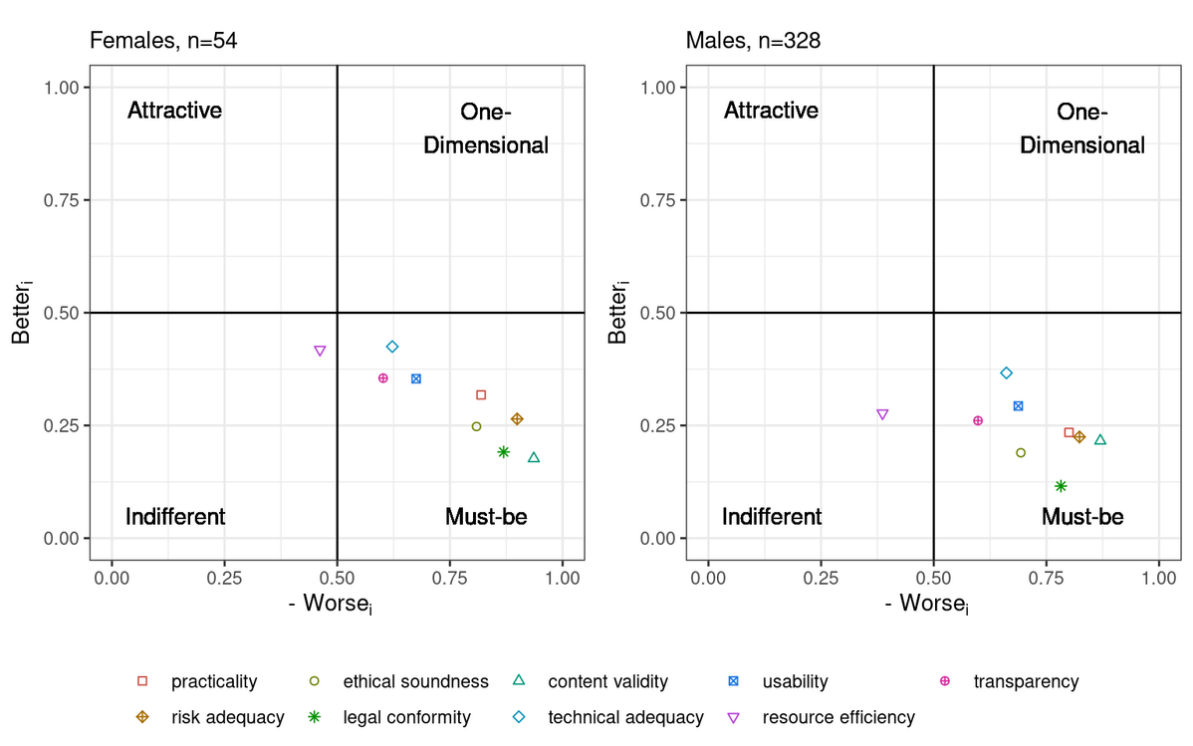
Plot of the Better and Worse coordinates per principle stratified by gender.

There was only a slight difference in the quality principle–related assessments between male and female participants. As there were too few female participants to prevent outliers from unduly influencing the results to continue evaluating groups A and B separately in this regard, the overall group of all participants was stratified by gender. There were only small differences in prioritization, despite (significant) disparities between both strata regarding the actual placement of the principles in the coordinate system (Figure 7 and Table 8).

**Table 8:** Ranking of the quality principles based on the distance of the Better and Worse coordinates to the outermost corner of the must-be quadrant, using the in-line-of-sight method for all participants, stratified by gender.

Quality principle	Coordinate distance between strata	Female participants					Male participants			
		Distance, *d*	Angle, *α*	Ranking coefficient, *f*	Rank	Distance, *d*		Angle, *α*	Ranking coefficient, *f*	Rank
Practicality	0.085	0.37	60	0.41	5	0.31		50	0.35	4
Risk adequacy	0.086	0.28	69	0.34	3	0.29		52	0.33	3
Ethical soundness	0.130	0.31	52	0.35	4	0.36		32	0.39	5
Legal conformity	0.115	0.23	56	0.28	2	0.25		28	0.27	1
Content validity	0.077	0.19	70	0.24	1	0.25		59	0.30	2
Technical adequacy	0.070	0.57	48	0.61	8	0.50		47	0.54	8
Usability	0.062	0.48	47	0.52	6	0.43		43	0.46	6
Resource efficiency	0.160	0.68	38	0.71	9	0.67		24	0.69	9
Transparency	0.094	0.53	42	0.57	7	0.48		33	0.51	7

#### Stratification by Interest in Digitization

**Figure 8:**
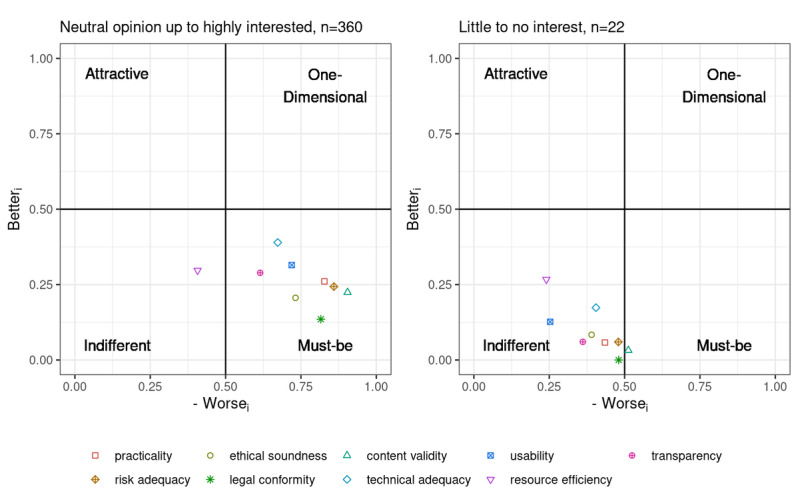
Plot of the Better and Worse coordinates per principle stratified by interest in the topic.

There were notable differences in ratings between those with a stated interest in digitization and those who lacked interest in this topic, again considering only the overall group and discarding groups A and B due to the low number of participants in the “little to no interest” stratum (Figure 8). For the latter group, the principles were almost exclusively located in the *indifferent* quadrant, or, in the case of legal conformity, content validity, and risk adequacy, near the border between the *indifferent* and *must-be* quadrants.

Nevertheless, the prioritization remained largely similar with that of the interest-based stratification, with only minor differences (see Table 9).

**Table 9:** Ranking of the quality principles based on the distance of the Better and Worse coordinates to the outermost corner of the must-be quadrant, using the in-line-of-sight method for all participants, stratified by their interest in digitization.

Quality principle	Coordinate distance between strata	Interested participants					Uninterested participants			
		Distance, *d*	Angle, *α*	Ranking coefficient, *f*	Rank	Distance, *d*		Angle, *α*	Ranking coefficient, *f*	Rank
Practicality	0.44	0.31	57	0.36	4	0.57		5.9	0.57	4
Risk adequacy	0.42	0.28	60	0.33	3	0.52		6.6	0.53	3
Ethical soundness	0.36	0.34	38	0.37	5	0.61		7.8	0.62	5
Legal conformity	0.36	0.23	36	0.26	1	0.52		0.0	0.52	2
Content validity	0.44	0.24	67	0.30	2	0.49		3.8	0.49	1
Technical adequacy	0.34	0.51	50	0.55	8	0.62		16.3	0.63	6
Usability	0.50	0.42	48	0.46	6	0.76		9.6	0.77	8
Resource efficiency	0.17	0.66	27	0.68	9	0.81		19.4	0.82	9
Transparency	0.34	0.48	37	0.51	7	0.64		5.4	0.65	7

## Discussion

### Principal Results

As shown in the literature (eg, [23,25,37,38]) as well as our own results, established methods for working with the results of Kano surveys are well-suited to determining generic user perceptions of product attributes of a health app, such as the quality principles that the participants of our survey were confronted with.

Nevertheless, when using Kano’s original approach, or even the more promising approach proposed by Timko [25] (with or without inclusion of the perceived relevance of the principles), in our case, the nine attributes remained firmly tethered to the *must-be* category (see Figure 6), with only resource efficiency crossing into the *indifferent* realm once perceived importance was included in the calculation. However, there were no *one-dimensional* or even *attractive* attributes. Solely based on established evaluation methods for Kano surveys, we therefore fell short of obtaining the desired ranking to be used for potentially prioritizing the health app quality principles.

Simply applying the Kano method and its categorizations to the quality principles initially did not allow for prioritization, which confirmed the previously noted similarity of the ratings [18,19], with again only resource efficiency standing out. As reported previously [18,19], the discrepancy between this quality principle and the other eight principles supports the assumption that resource efficiency likely only plays a minor role in today’s mostly very powerful devices, since health-related apps in particular presumably place little demand on the devices.

To counteract this lack of differentiation between the principles, we then developed the so-called “in-line-of-sight” method, which, based on the numeric values representing satisfaction as well as dissatisfaction with the respective attribute or quality principle, determines a ranking coefficient while also accounting for different points of view (depending on the purpose of the desired prioritization). This method should also be flexible enough to be adapted to different circumstances depending on the use case and user ratings provided.

In our exemplary evaluation for the ranking from the *must-be* perspective, we chose a rather conservative approach, factoring in an angle that leads to lower Worse values being preferred, while accepting that by choosing this approach, the values for Better will also decrease.

This corresponds to the definition of the *must-be* category: a lack of the respective quality principle would be perceived more strongly than the positive effect that would be achieved if the characteristics consistent with the quality principle were present. When changing the perspective to another quadrant, similar considerations need to be applied, with calculations being adapted accordingly. For example, when changing the perspective to the *attractive* quadrant, it would be more useful to aim at a higher priority of Better values, as this better represents the definition of this category.

### Kano Survey Interpretation: Potential for Linguistic Inconsistencies?

Although the Kano model is popular and is often used in a wide variety of contexts, linguistic inaccuracies in its application have arisen over the years, which in some publications have led to difficulties in its correct application or to supposed inconsistencies ([29], citing [25]). The problem originates from an inaccurate translation of Kano’s key concept transliterated as “atarimae,” which has been translated as *must-be* in many English-language publications. *Must-be* seems to have first been used in the early 1990s by Shoji Shiba when presenting the Kano model to English-speaking audiences [29]. However, apparently, the meaning of “atarimae” would be better represented by the terms “natural,” “obvious,” “expected,” “ordinary,” or “normal.” This change should be applied to the category name *must-be* as well as the corresponding customer response, which is often given as “It must be that way,” but, as noted by Horton and Goers [29], should rather be represented by translations along the lines of the aforementioned suggestions.

When Kano surveys are translated into other languages, this inaccuracy may be passed on to a varying degree, potentially further complicating the situation. In our (German language) questionnaire, however, we already included the wording representing “I take this for granted” (German: “Setze ich voraus”) as an answer option for the participants instead of *must-be*, thus more closely following Kano’s original idea. To stay in line with most of the literature, we nevertheless decided to stick to the *must-be* term, although this aspect needs to be kept in mind. This change in interpretation may also provide an explanation for the results we obtained for the nine quality principles, with all of them being located in the *must-be* category.

In contrast to common usage scenarios for Kano surveys that aim at selecting attributes one should further investigate, we applied the model to a set of attributes, namely our quality principles, that had already been painstakingly compiled [16,17] (among others based on various norms (eg, [3,39-42]), as well as the literature (eg, [5,6,43,44]). This may provide an additional explanation for why, in the survey presented here, all quality principles were rated as *must-be*, or following the adapted interpretation, as “obvious” or “something to be taken for granted.” That is, the quality principles simply followed obvious requirements that were mentioned as essential in the aforementioned sources, and that one would expect users to be able to rate objectively (at least to a certain degree); they were, however, not selected in order to trigger enthusiasm. Their placement in the *must-be* quadrant is therefore easily explained, and the sole exception for resource efficiency being placed in the *indifferent* category may possibly be due to the fact that today’s mobile devices are commonly equipped with sufficient computing power—at least for physicians, who often probably have access to rather high-end devices—so that resources are not a factor that warrants considerable attention.

### Selection of the Evaluation Method Used as a Basis of this Work

In addition to the linguistic aspects, there is no clear verdict about the methodology one should apply foremost when evaluating Kano model–based surveys. While there is a large variety of methods to choose from, based on various theoretical concepts, the discussion is still open as to which of them is most appropriate (in general or for a specific use case) and has the greatest validity. Although there are various empirical evaluations of different approaches in the context of Kano surveys that are described in the literature (eg, [37,38,45-47]), determining which of these particular approaches is best seems to be near impossible.

As stated by Mikulić and Prebezac [23], the validity and reliability of the various approaches cannot be determined with certainty: there is simply no known comparison that can be taken as the ground truth.

Which method is chosen is therefore rather often a matter of whether (1) the theoretical justification of the respective approach appears valid, (2) the increase in information when applying the respective approach actually contributes to the solution of the problem, and (3) which (recognizable) technical strengths and weaknesses the approach has.

For the purposes of this paper, Timko’s approach (first introduced in [25]) was therefore chosen as a foundation, as it is easy to understand and also easily allows for integration of the self-stated relevance of the attributes to be evaluated. Additionally, compared to Kano’s initial idea, where, essentially, all 25 possible answer combinations are directly mapped to only 6 possible categories, one may feel the need for a more differentiated, continuous method of analyzing the data to better assess how different attributes are similar or dissimilar, and our enhanced approach follows this line of thought.

### Limitations

#### Selection of the Quality Principles Employed in This Study

New information technologies, including online information or specific (mobile) apps, place additional demands on those employing them, especially in professional health care contexts. Professionals employing such technologies need to ensure that they are safe and pose no harm to those in their care. Regulatory oversight as well as evidence-based literature are often found lacking [48]. Economic questions such as the paucity of information related to cost-effectiveness or cost-utility [49,50], or even aspects related to reimbursement [51,52] may also play a role in whether or not the technologies are actually adopted in everyday practice.

Without at least a basic understanding of the relevant quality aspects (and how to apply them), or uncertainties regarding their safety and security, acceptance may suffer, which may also limit the potential of these technologies [48,53]. However, there is no general consensus, even among experts, as to what exactly constitutes “quality” in this context and how it can be assessed for specific scenarios (eg, to rate health-related apps) [54].

To identify items of relevance, such as for inclusion in various tools [4-8] meant to aid in assessing such technologies that are to be provided to the respective target groups (eg, physicians or other health care personnel), it is important to identify certain key aspects in the hope that these fulfill the information needs and information-seeking behaviors of users [55]. Many authors use rather detailed approaches and criteria to enable this information-seeking and more easily assess the quality of health-related apps, and they often target specific (professional) user groups [54].

For this purpose, in close collaboration with various stakeholders (eg, experts convened on behalf of eHealth Suisse), the nine quality principles used here were compiled [16,17] and evaluated [18,19]. In this context, we were able to show that, despite its broad scope and lack of details, and being almost unanimously regarded as (highly) relevant by the participants of both previous studies, the predefined set of quality principles was still well-suited to provide the respective participants with pointers to aspects relevant for determining an app’s quality and fine-tuning their usage decisions. After having been sensitized to the topic of quality principles, and having applied these principles to exemplary app descriptions, the participants of both previous studies were able to make a much more differentiated assessment of the app descriptions that were provided, and were much more critical in their decision on whether or not to potentially use the corresponding app.

#### Survey Design

Although we had initially considered an additional qualitative approach, specifically to ask the participants to directly rank the principles as they saw fit, a major reason that made us abandon this course of action was that the data presented here were part of a larger project (as mentioned above, the first part of the analysis of the acquired data is already published [28]), and it was decided by the team that an additional (sorting) questionnaire would be too much of a burden for those participating in the survey. Of the two alternatives for designing the part of the survey presented here (ie, continuing to rely on the Kano model or using the qualitative sorting approach), the choice ultimately fell on Kano. This was based on our hope to be able to use the data obtained for implicit assessment instead of running the risk that the previously established, highly similar assessments of the principles would make it difficult for the participants to determine a specific order. Because we did not initially know how many people would participate, we were concerned that it would be difficult to determine an overall ranking for the nine principles if too few people participated and we only relied on the explicitly stated rankings. It was hoped that based on Kano’s methods, using the provided answers and ensuing categorizations, we would be able to at least determine a rough prioritization for the overall group of participants, in our case, by giving principles in the *must-be* or *one-dimensional* categories precedence over those in the *attractive* or *indifferent* categories.

#### Study Participants

Despite having contacted a relatively large number of potential participants, with only 4.02% (382/9503) of those who were initially invited actually completing the survey, the response rate was low. Based on this response rate and demographic factors, the results, specifically those related to any rankings of attributes presented here, may not be fully representative of physicians overall or even those specializing in orthopedic or trauma surgery.

One of the possibly most relevant demographic factors for which one might potentially expect an impact on the assessments is the gender of the participants. Overall, the gender distribution of the participants roughly corresponded to the ratio expected in orthopedics. In our survey, 85.9% (328/382) of the participants were male and 14.1% (54/382) were female. Thus, there were only slightly fewer women than would have been expected in the field of orthopedics and trauma surgery, according to data provided by the Bundesärztekammer, with 17.63% (3611/20,477), as of December 31, 2020, of those in the fields of orthopedics or orthopedics and trauma surgery being women [56].

However, gender seems to only have exerted a limited influence on prioritization, which is in line with our previous work [18,19], where there were also only minor differences in the quality principle–related assessments between male and female participants. Differences were particularly pronounced for resource efficiency and ethical soundness (see the column describing the coordinate distance between both strata in Table 8, as well as Figure 7 for the actual coordinates). The former was placed near the (neutral) center for female participants, whereas for male participants, it was clearly placed in the *indifferent* quadrant. Content validity and usability (along with transparency for female participants) were somewhat closer to the *one-dimensional* quadrant than the other principles in both strata. In case of the female participants, the point cloud was also shifted more toward the *one-dimensional* quadrant compared with that of their male peers, and the coordinates were less scattered overall (Figure 7).

Regarding the ranking of the principles, for the female participants (n=54), content validity ranked first and legal conformity ranked second (Table 8). For the male (n=328) participants, this order was reversed. The same was true for ethical soundness and practicality. Apart from resource efficiency, all quality principles were found in the *must-be* quadrant (Figure 7).

Nevertheless, the prioritization was roughly similar for the two demographic groups: for the female participants (n=54), content validity ranked first and legal conformity was placed second (Table 8), whereas this order was reversed for the male (n=328) participants. The same was true for ethical soundness and practicality. Apart from resource efficiency, all quality principles were found in the *must-be* quadrant (Figure 7).

Considering interest in digitization (Figure 8 and Table 9), digitally affine participants (aggregated data for “neutral,” “interested,” or “highly interested”; n=360) were considerably overrepresented due to the chosen survey method. Participants with little interest in the topic, or those lacking access to the techniques used, responded much less frequently than those showing more enthusiasm toward digitization, thus potentially biasing the results as well. However, for the limited number of participants (n=22) who cared only little about digitization (values aggregated for being “less interested” or “not interested” in the topic), but nevertheless participated, it was primarily the placement of the points representing the quality principles in the coordinate system that differed strikingly from the other participants (Figure 8). There was also a striking difference in the placement of the principles within the coordinate system, which is probably not solely attributable to the imbalance between the sizes of the two groups. Disinterested participants rated the principles as *indifferent*, or, in the case of legal conformity, content validity, and risk adequacy, near the border between the *indifferent* and *must-be* quadrants (Figure 8). Nevertheless, rankings remained largely similar independent of digital affinity. For those stating a more or less pronounced interest into digitalization, the order of practicality and risk adequacy was reversed compared with that of the participants with little to no interest. Among disinterested participants, there were also small deviations in the rank for legal conformity and content validity (reverse rank 1 and 2, respectively) as well as technical adequacy and usability (rank 6 and 8, respectively; see Table 9). Legal conformity, content validity, and risk adequacy occupied the top ranks among participants with or without interest, but the order for content validity and legal conformity differed. The lower ranks were occupied by usability, transparency, technical adequacy, and resource efficiency, albeit with a somewhat differing order.

The difference in locations of the principles in the coordinate system (Figure 8), but not in the prioritizations obtained for the two groups (Table 9), lends support to the feasibility of applying our method to quality principles in the mHealth app domain, and supports the need for better education of (potential) users of mHealth apps. Although medical professionals such as our participants are—or at any rate should be—aware of the need for quality (as demanded by professional ethics) for all tools they apply in care contexts, it seems as though for those lacking interest in digitization, this mental transfer apparently does not work for the uninterested participants, as shown by their indifferent ratings. Educational campaigns such as those by professional societies that emphasize the need for quality not only in conventional care but also in the digital domain, including mHealth apps, may help to raise awareness in this regard even for those who are not (yet) familiar or comfortable with the use of such technologies in their daily work.

Altogether, an additional, hopefully larger-scale, study should be implemented to obtain more conclusive data for these as well as other demographic strata, such as by recruiting additional participants with the aid of other professional organizations or by including additional target groups such as patient organizations, universities providing medical education, and others.

#### Implementation

We believe to have found a methodology that is well-adapted to the demands of finding a prioritization of app quality principles in the case of very similar categorizations, clustered in either of the four categories of *must-be*, *one-dimensional*, *attractive*, or *indifferent* obtained using a Kano questionnaire.

Of course, our method needs further validation, and, depending on the scenario in which it is applied, it might be helpful to adapt the strategy of how the angles (or their direction) are calculated. This may depend on multiple factors. For example, when considering ratings based on *must-be*, it seems sensible to always perform sorting based on the distance to the *one-dimensional* rather than to the *indifferent* quadrant, as an *indifferent* opinion, per se, does not elicit identification with the product (or its attributes).

However, if one switches perspective to the *one-dimensional* category, it may well depend on the type of product, its application areas, as well as its target user group, along with the attributes actually being evaluated if it makes more sense to calculate the angles used in determining the sorting against *must-be* or *attractive*. For products targeting professionals, it might, for example, make more sense to sort the quality principles depending on their closeness to *must-be*, whereas for marketing purposes, *attractive* qualities may be more promising. Again, for the *attractive* corner, similar arguments as for the approach taken for *must-be* apply, with the angle toward *one-dimensional* rather than *indifferent,* which likely makes more sense in most scenarios.

If attributes were clustered in the *indifferent* corner, the question of the direction to base any attribute sorting on is again more open (ie, toward either *attractive* or *must-be*). The decision may also depend somewhat on the purpose, design, and area of use of the product under consideration; in the case of a professional product, it may potentially make more sense to build the ranking based on *must-be* as a reference, since *attractiveness* does not necessarily reflect professional quality.

### Outlook and Comparison With Previous Work

Further proof of the validity of the method and its transferability to other interest groups, quality attributes, or application scenarios is still pending. Future work will particularly have to address further validation of the method with regard to the evaluation involving other user groups (eg, patients, caregivers) or to the application for prioritization of other attributes, whether for use in medical or general apps, or for the evaluation of other attribute lists outside the app domain.

**Figure 9:**
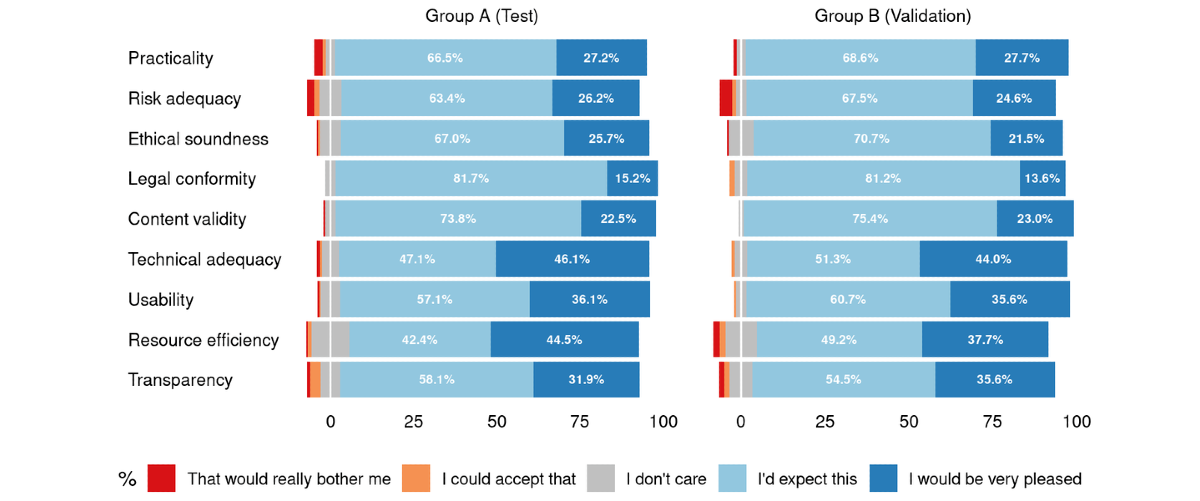
Relevance ratings for the nine quality principles: comparison between this survey and previously published work [19]. See Table S4 in Multimedia Appendix 1 for the corresponding *P* values of this comparison. DGIM: German Association for Internal Medicine, German: "Deutsche Gesellschaft für Innere Medizin e.V.".

However, especially with regard to the determined ranking of the quality criteria we chose for this evaluation, we believe that a comparison of the perception of relevance between the results of the previous studies (eg, [19], where participants were working in a different medical field) and those shown here is a strong indicator that the results are likely transferable. Similar to the current work, participants of previous studies had also been asked to provide their opinion regarding the relevance of the nine quality principles, and the participating physicians rated the relevance of the quality principles similar to the current group of participants (see Figure 9), with only minor (and statistically negligible) differences between the previous study [19] and the data obtained from the participants of this study. Table S4 in Multimedia Appendix 1 shows the overall relevance ratings and *P* values for the comparison between the two studies. However, for the sake of streamlining the comparison between studies, the respective test and validation samples, as they were used in both studies, were aggregated. Similarly, to stay in line with Albrecht et al [19], the answer options for “very important” and “important” were summarized using the term “important,” while those for “less important” and “unimportant” were aggregated as “not important.”

As shown in Figure 9 and Table S4 in Multimedia Appendix 1, there are notable similarities between both studies: the proportion of participants that rated *resource efficiency* as important was decidedly lower (current study: 260/382, 68.1% participants; previous study: 270/441, 61.6%) than it was for all other quality principles, where the perceived importance was in the range of 84%-98%, again for both studies.

### Conclusions

The agreement with respect to perceived relevance between both studies, as shown above, leads to the following conclusions.

For both previous studies [18,19], there was no clear pathway for prioritization of the principles should the need arise, apart from resource efficiency consistently being the least popular quality principle, with ensuing lesser relevance. However, in today’s medical world, time is a valuable commodity, and in fact, a lack of time or too much effort being required to adequately assess all relevant aspects is often mentioned as a barrier both to accessing information [55] as well as to employing apps in specific situations (eg, for consultations [57]). Although health apps may initially give the impression of being able to save time and reduce effort, professional ethics (eg, [58,59]) demand that those working in medical professions must ensure that any (digital) tools they use are up to the expected professional standards. In the digital world, even aided by various tools meant to aid in the process, health care professionals often remain unsure of which factors they need to consider in this context, especially if the tools require extensive effort. This may possibly contribute to the many—real or perceived—barriers toward successfully using apps in care settings or for health-related purposes in general.

Of course, an all-encompassing, unaided, and professionally conducted evaluation of apps will neither be possible nor practical in most scenarios, largely due to a lack of technical expertise. However, physicians and other health care professionals should at least be enabled to assess available information in the context of their work, such as based on a set of questions [19] that address basic quality principles. Even for such limited lists, being able to determine a ranking of the questions or quality principles seems sensible for assessing highly available information with priority; if the initially evaluated factors already lead to a rejection, the remaining factors can justifiably be disregarded, thus saving the time that a full, structured assessment based on such questions covering all available information sources (eg, from the app store, on manufacturer websites, and other sources) would take. For longer lists of quality principles or rating criteria applied to mHealth apps, the benefits of being able to determine a sensible and context-adapted prioritization, based on feedback obtained from the respective peer group, may be even greater, counteracting or at least somewhat alleviating arguments that many of the available rating tools or quality principles are—due to the large number of details they cover—too cumbersome for real-word applications outside of academic evaluations [22].

In contrast to other approaches based on the Kano method (eg, [23,25]) that predominantly strive for categorization of the attributes being evaluated, the methodology presented here may provide an interesting option that additionally allows for the prioritization of quality principles in cases of largely similar categorization results or initial user perceptions. This may aid in giving precedence to the most relevant (prioritized) principles, deferring those with lesser priority. To what extent the method will be applicable beyond the usage scenario described here will require more extensive investigations.

However, it also remains an open question as to how one could deal with cases where for a larger number of attributes, there are multiple close clusters of attributes found in different quadrants. One possible solution to this might be to sort attributes in each cluster as described above, and to then perform a prioritization of the clusters themselves (with attributes in the *attractive* quadrant probably being the most relevant) in order to arrive at a full ranking of all attributes to be considered.

Nevertheless, the proposed prioritization may provide a means for professional organizations that want to give their members a recommendation as to which quality principles should be applied with priority in digital domains, independent of whether this is done for the generic set of app-related quality principles or principles that are more subject-specific (eg, for use in a particular medical specialty or for a specific user group).

## Appendix

Multimedia Appendix 1 Additional tables.

## Abbreviations

BVOU: Berufsverband für Orthopädie und Unfallchirurgie (Professional Association for Orthopedics and Trauma Surgery)
DGOU: Deutsche Gesellschaft für Orthopädie und Unfallchirurgie (German Society for Orthopedics and Trauma Surgery)
DVG: German Digital Healthcare Act
mHealth: mobile health
