# Supplementary material for: Prioritization of Quality Principles for Health Apps Using the Kano Model: Survey Study
Source: JMIR Mhealth Uhealth. 2022 Jan 11;10(1):e26563. doi: 10.2196/26563 (PMC8790690; doi:10.2196/26563)
Supplement: Multimedia Appendix 1 [file mhealth_v10i1e26563_app1.pdf]

Multimedia Appendix 1: Additional Tables

Table S1: Answers to the functional questions (raw data, per quality principle).

Answers to the functional questions per quality principle	A, N=191: n (%)	B, N=191: n (%)	Total, N=382: n (%)	*P* value
**Practicality**				0.500[1]
I would be very pleased	52 (27.2%)	53 (27.7%)	105 (27.5%)	
I'd expect this	127 (66.5%)	131 (68.6%)	258 (67.5%)	
I don't care	5 (2.6%)	5 (2.6%)	10 (2.6%)	
I could accept that	2 (1.0%)	0 (0.0%)	2 (0.5%)	
That would really bother me	5 (2.6%)	2 (1.0%)	7 (1.8%)	
**Risk adequacy**				0.413[1]
I would be very pleased	50 (26.2%)	47 (24.6%)	97 (25.4%)	
I'd expect this	121 (63.4%)	129 (67.5%)	250 (65.4%)	
I don't care	13 (6.8%)	6 (3.1%)	19 (5.0%)	
I could accept that	3 (1.6%)	2 (1.0%)	5 (1.3%)	
That would really bother me	4 (2.1%)	7 (3.7%)	11 (2.9%)	
**Ethical soundness**				0.726[1]
I would be very pleased	49 (25.7%)	41 (21.5%)	90 (23.6%)	
I'd expect this	128 (67.0%)	135 (70.7%)	263 (68.8%)	
I don't care	12 (6.3%)	14 (7.3%)	26 (6.8%)	
I could accept that	1 (0.5%)	0 (0.0%)	1 (0.3%)	
That would really bother me	1 (0.5%)	1 (0.5%)	2 (0.5%)	
**Legal conformity**				0.356[1]
I would be very pleased	29 (15.2%)	26 (13.6%)	55 (14.4%)	
I'd expect this	156 (81.7%)	155 (81.2%)	311 (81.4%)	
I don't care	6 (3.1%)	7 (3.7%)	13 (3.4%)	
I could accept that	0 (0.0%)	3 (1.6%)	3 (0.8%)	
**Content validity**				0.564[1]
I would be very pleased	43 (22.5%)	44 (23.0%)	87 (22.8%)	
I'd expect this	141 (73.8%)	144 (75.4%)	285 (74.6%)	
I don't care	6 (3.1%)	3 (1.6%)	9 (2.4%)	
That would really bother me	1 (0.5%)	0 (0.0%)	1 (0.3%)	
**Technical adequacy**				0.510[1]
I would be very pleased	88 (46.1%)	84 (44.0%)	172 (45.0%)	
I'd expect this	90 (47.1%)	98 (51.3%)	188 (49.2%)	
I don't care	10 (5.2%)	7 (3.7%)	17 (4.5%)	
I could accept that	1 (0.5%)	2 (1.0%)	3 (0.8%)	
That would really bother me	2 (1.0%)	0 (0.0%)	2 (0.5%)	

Answers to the functional questions per quality principle	A, N=191: n (%)	B, N=191: n (%)	Total, N=382: n (%)	*P* value
**Usability**				0.610[1]
I would be very pleased	69 (36.1%)	68 (35.6%)	137 (35.9%)	
I'd expect this	109 (57.1%)	116 (60.7%)	225 (58.9%)	
I don't care	11 (5.8%)	6 (3.1%)	17 (4.5%)	
I could accept that	1 (0.5%)	1 (0.5%)	2 (0.5%)	
That would really bother me	1 (0.5%)	0 (0.0%)	1 (0.3%)	
**Resource efficiency**				0.349[1]
I would be very pleased	85 (44.5%)	72 (37.7%)	157 (41.1%)	
I'd expect this	81 (42.4%)	94 (49.2%)	175 (45.8%)	
I don't care	22 (11.5%)	18 (9.4%)	40 (10.5%)	
I could accept that	2 (1.0%)	3 (1.6%)	5 (1.3%)	
That would really bother me	1 (0.5%)	4 (2.1%)	5 (1.3%)	
**Transparency**				0.740[1]
I would be very pleased	61 (31.9%)	68 (35.6%)	129 (33.8%)	
I'd expect this	111 (58.1%)	104 (54.5%)	215 (56.3%)	
I don't care	11 (5.8%)	13 (6.8%)	24 (6.3%)	
I could accept that	6 (3.1%)	3 (1.6%)	9 (2.4%)	
That would really bother me	2 (1.0%)	3 (1.6%)	5 (1.3%)	

[1]Pearson's Chi-squared test

Table S2: Answers to the dysfunctional questions (raw data, per quality principle).

Answers to the dysfunctional questions for…	A, N=191: n (%)	B, N=191: n (%)	Total, N=382: n (%)	*P* value
**Practicality**				0.160[1]
I would be very pleased	0 (0.0%)	2 (1.0%)	2 (0.5%)	
I'd expect this	0 (0.0%)	3 (1.6%)	3 (0.8%)	
I don't care	7 (3.7%)	9 (4.7%)	16 (4.2%)	
I could accept that	12 (6.3%)	7 (3.7%)	19 (5.0%)	
That would really bother me	172 (90.1%)	170 (89.0%)	342 (89.5%)	
**Risk adequacy**				0.647[1]
I would be very pleased	0 (0.0%)	2 (1.0%)	2 (0.5%)	
I'd expect this	2 (1.0%)	3 (1.6%)	5 (1.3%)	
I don't care	8 (4.2%)	6 (3.1%)	14 (3.7%)	
I could accept that	3 (1.6%)	3 (1.6%)	6 (1.6%)	
That would really bother me	178 (93.2%)	177 (92.7%)	355 (92.9%)	

**Answers to the dysfunctional questions for…**	**A, N=191: n (%)**	**B, N=191: n (%)**	**Total, N=382: n (%)**	***P* value**
**Ethical soundness**				0.975[1]
I would be very pleased	1 (0.5%)	1 (0.5%)	2 (0.5%)	
I'd expect this	3 (1.6%)	4 (2.1%)	7 (1.8%)	
I don't care	12 (6.3%)	11 (5.8%)	23 (6.0%)	
I could accept that	15 (7.9%)	18 (9.4%)	33 (8.6%)	
That would really bother me	160 (83.8%)	157 (82.2%)	317 (83.0%)	
**Legal conformity**				0.364[1]
I would be very pleased	0 (0.0%)	2 (1.0%)	2 (0.5%)	
I'd expect this	1 (0.5%)	3 (1.6%)	4 (1.0%)	
I don't care	6 (3.1%)	6 (3.1%)	12 (3.1%)	
I could accept that	9 (4.7%)	14 (7.3%)	23 (6.0%)	
That would really bother me	175 (91.6%)	166 (86.9%)	341 (89.3%)	
**Content validity**				0.097[1]
I would be very pleased	0 (0.0%)	1 (0.5%)	1 (0.3%)	
I'd expect this	2 (1.0%)	2 (1.0%)	4 (1.0%)	
I don't care	7 (3.7%)	4 (2.1%)	11 (2.9%)	
I could accept that	0 (0.0%)	6 (3.1%)	6 (1.6%)	
That would really bother me	182 (95.3%)	178 (93.2%)	360 (94.2%)	
**Technical adequacy**				0.823[1]
I would be very pleased	2 (1.0%)	2 (1.0%)	4 (1.0%)	
I'd expect this	0 (0.0%)	1 (0.5%)	1 (0.3%)	
I don't care	10 (5.2%)	8 (4.2%)	18 (4.7%)	
I could accept that	28 (14.7%)	32 (16.8%)	60 (15.7%)	
That would really bother me	151 (79.1%)	148 (77.5%)	299 (78.3%)	
**Usability**				0.451[1]
I would be very pleased	0 (0.0%)	2 (1.0%)	2 (0.5%)	
I'd expect this	2 (1.0%)	3 (1.6%)	5 (1.3%)	
I don't care	11 (5.8%)	6 (3.1%)	17 (4.5%)	
I could accept that	25 (13.1%)	25 (13.1%)	50 (13.1%)	
That would really bother me	153 (80.1%)	155 (81.2%)	308 (80.6%)	
**Resource efficiency**				0.053[1]
I would be very pleased	0 (0.0%)	4 (2.1%)	4 (1.0%)	
I'd expect this	1 (0.5%)	5 (2.6%)	6 (1.6%)	
I don't care	24 (12.6%)	14 (7.3%)	38 (9.9%)	
I could accept that	63 (33.0%)	62 (32.5%)	125 (32.7%)	
That would really bother me	103 (53.9%)	106 (55.5%)	209 (54.7%)	

Answers to the dysfunctional questions for…	A, N=191: n (%)	B, N=191: n (%)	Total, N=382: n (%)	*P* value
**Transparency**				0.526[1]
I would be very pleased	2 (1.0%)	1 (0.5%)	3 (0.8%)	
I'd expect this	1 (0.5%)	4 (2.1%)	5 (1.3%)	
I don't care	13 (6.8%)	15 (7.9%)	28 (7.3%)	
I could accept that	29 (15.2%)	35 (18.3%)	64 (16.8%)	
That would really bother me	146 (76.4%)	136 (71.2%)	282 (73.8%)	

[1]Pearson's Chi-squared test

Table S3: Perceived relevance for the nine quality criteria (raw data).

Perceived relevance	A, N=191: n (%)	B, N=191: n (%)	Total, N=382: n (%)	*P* value
**Practicality**				0.891[1]
unimportant	6 (3.1%)	7 (3.7%)	13 (3.4%)	
less important	3 (1.6%)	1 (0.5%)	4 (1.0%)	
neutral	5 (2.6%)	5 (2.6%)	10 (2.6%)	
important	48 (25.1%)	50 (26.2%)	98 (25.7%)	
very important	129 (67.5%)	128 (67.0%)	257 (67.3%)	
**Risk adequacy**				0.056[1]
unimportant	5 (2.6%)	4 (2.1%)	9 (2.4%)	
less important	0 (0.0%)	2 (1.0%)	2 (0.5%)	
neutral	11 (5.8%)	2 (1.0%)	13 (3.4%)	
important	55 (28.8%)	65 (34.0%)	120 (31.4%)	
very important	120 (62.8%)	118 (61.8%)	238 (62.3%)	
**Ethical soundness**				0.387[1]
unimportant	7 (3.7%)	4 (2.1%)	11 (2.9%)	
less important	5 (2.6%)	9 (4.7%)	14 (3.7%)	
neutral	14 (7.3%)	16 (8.4%)	30 (7.9%)	
important	46 (24.1%)	57 (29.8%)	103 (27.0%)	
very important	119 (62.3%)	105 (55.0%)	224 (58.6%)	
**Legal conformity**				0.079[1]
unimportant	4 (2.1%)	5 (2.6%)	9 (2.4%)	
less important	2 (1.0%)	5 (2.6%)	7 (1.8%)	
neutral	11 (5.8%)	5 (2.6%)	16 (4.2%)	
important	41 (21.5%)	60 (31.4%)	101 (26.4%)	
very important	133 (69.6%)	116 (60.7%)	249 (65.2%)	

Perceived relevance	A, N=191: n (%)	B, N=191: n (%)	Total, N=382: n (%)	*P* value
**Content validity**				0.330[1]
unimportant	5 (2.6%)	3 (1.6%)	8 (2.1%)	
less important	2 (1.0%)	0 (0.0%)	2 (0.5%)	
neutral	2 (1.0%)	0 (0.0%)	2 (0.5%)	
important	35 (18.3%)	37 (19.4%)	72 (18.8%)	
very important	147 (77.0%)	151 (79.1%)	298 (78.0%)	
**Technical adequacy**				0.717[1]
unimportant	5 (2.6%)	4 (2.1%)	9 (2.4%)	
less important	3 (1.6%)	4 (2.1%)	7 (1.8%)	
neutral	13 (6.8%)	17 (8.9%)	30 (7.9%)	
important	80 (41.9%)	68 (35.6%)	148 (38.7%)	
very important	90 (47.1%)	98 (51.3%)	188 (49.2%)	
**Usability**				0.317[1]
unimportant	7 (3.7%)	3 (1.6%)	10 (2.6%)	
less important	1 (0.5%)	4 (2.1%)	5 (1.3%)	
neutral	12 (6.3%)	10 (5.2%)	22 (5.8%)	
important	69 (36.1%)	80 (41.9%)	149 (39.0%)	
very important	102 (53.4%)	94 (49.2%)	196 (51.3%)	
**Resource efficiency**				0.310[1]
unimportant	11 (5.8%)	3 (1.6%)	14 (3.7%)	
less important	18 (9.4%)	19 (9.9%)	37 (9.7%)	
neutral	35 (18.3%)	36 (18.8%)	71 (18.6%)	
important	75 (39.3%)	77 (40.3%)	152 (39.8%)	
very important	52 (27.2%)	56 (29.3%)	108 (28.3%)	
**Transparency**				0.358[1]
unimportant	8 (4.2%)	4 (2.1%)	12 (3.1%)	
less important	3 (1.6%)	8 (4.2%)	11 (2.9%)	
neutral	17 (8.9%)	22 (11.5%)	39 (10.2%)	
important	82 (42.9%)	78 (40.8%)	160 (41.9%)	
very important	81 (42.4%)	79 (41.4%)	160 (41.9%)	

[1]Pearson's Chi-squared test

Table S4: Comparison of relevance ratings for the nine quality principles between the survey study presented here (answers provided by members of the DGOU) and previously published work (members of the DGIM [19]).

**Quality principle**	**Current study (DGOU), N=382: n (%)**	**Previous study (DGIM), N=441: n (%)**	***P* value**
**Practicality**			0.307
not important	17 (4.5%)	22 (5.0%)	
neutral	10 (2.6%)	31 (7.0%)	
important	355 (92.9%)	379 (85.9%)	
not answered	0 (0.0%)	9 (2.0%)	
**Risk adequacy**			0.900
not important	11 (2.9%)	6 (1.4%)	
neutral	13 (3.4%)	30 (6.8%)	
important	358 (93.7%)	402 (91.2%)	
not answered	0 (0.0%)	3 (0.7%)	
**Ethical soundness**			0.887
not important	25 (6.5%)	24 (5.4%)	
neutral	30 (7.9%)	45 (10.2%)	
important	327 (85.6%)	369 (83.7%)	
not answered	0 (0.0%)	3 (0.7%)	
**Legal conformity**			0.626
not important	16 (4.2%)	14 (3.2%)	
neutral	16 (4.2%)	23 (5.2%)	
important	350 (91.6%)	402 (91.2%)	
not answered	0 (0.0%)	2 (0.5%)	
**Content validity**			0.041
not important	10 (2.6%)	2 (0.5%)	
neutral	2 (0.5%)	6 (1.4%)	
important	370 (96.9%)	432 (98.0%)	
not answered	0 (0.0%)	1 (0.2%)	
**Technical adequacy**			0.707
not important	16 (4.2%)	13 (2.9%)	
neutral	30 (7.9%)	54 (12.2%)	
important	336 (88.0%)	371 (84.1%)	
not answered	0 (0.0%)	3 (0.7%)	

**Quality principle**	**Current study (DGOU), N=382: n (%)**	**Previous study (DGIM), N=441: n (%)**	***P* value**
**Usability**			0.360
not important	15 (3.9%)	17 (3.9%)	
neutral	22 (5.8%)	41 (9.3%)	
important	345 (90.3%)	381 (86.4%)	
not answered	0 (0.0%)	2 (0.5%)	
**Resource efficiency**			0.382
not important	51 (13.4%)	54 (12.2%)	
neutral	71 (18.6%)	114 (25.9%)	
important	260 (68.1%)	270 (61.2%)	
not answered	0 (0.0%)	3 (0.7%)	
**Transparency**			0.127
not important	23 (6.0%)	14 (3.2%)	
neutral	39 (10.2%)	53 (12.0%)	
important	320 (83.8%)	367 (83.2%)	
not answered	0 (0.0%)	7 (1.6%)	
